# Time Spent in Sedentary Behaviour as Discriminant Criterion for Frailty in Older Adults

**DOI:** 10.3390/ijerph15071336

**Published:** 2018-06-26

**Authors:** Venicius Dantas da Silva, Sheilla Tribess, Joilson Meneguci, Jeffer Eidi Sasaki, Douglas de Assis Teles Santos, José Ailton Oliveira Carneiro, Jair Sindra Virtuoso Júnior

**Affiliations:** 1 Center for Research in Physical Activity & Health, Federal University of Triangulo Mineiro, Uberaba MG 38061-500, Brazil; veniciusdantas@hotmail.com (V.D.d.S.); sheillatribess@yahoo.com.br (S.T.); joilsonmeneguci@yahoo.com.br (J.M.); jeffersasaki@gmail.com (J.E.S.); 2 Department of Education, State University of Bahia, Teixeira de Freitas 45992-255, Brazil; datsantos@uneb.br; 3 Department of Health, Southwestern State University of Bahia, Jequié 45206-190, Brazil; hitoef@yahoo.com.br

**Keywords:** sedentary behaviour, frailty, roc curve

## Abstract

This paper aims to analyse whether time spent in sedentary behaviour was a discriminant criterion for frailty in older adults. This was a cross-sectional study conducted in a sample of 457 elderly individuals aged ≥60 years. Frailty was defined as the presence of three or more of the following criteria: Unintentional weight loss, low walking speed at a 4.57 m course, reduced manual grip strength, exhaustion and insufficient physical activity level. Participants were classified into two groups: Non-frail or frail. Exposure to sedentary behaviour was assessed by the time spent sitting during a typical week, according to the adapted version of the International Physical Activity Questionnaire. Descriptive (mean, frequency) and inferential statistics (Poisson regression, Pearson’s Chi-square, Receiver Operating Characteristic Curve) were used to analyse the data, comparing them to the time-related areas exposed to sedentary behaviour by gender and the presence of fragility. The prevalence of frailty was 22.1% (*n* = 101). The most accurate cut-off points of sitting time for predicting frailty were >495 min/day (men) or >536 min/day (women). Time spent in sedentary behaviour can be used to indicate fragility in the elderly of both sexes.

## 1. Introduction

Frailty is a negative state of health that does not present a concise definition in relation to its characterization. Experts in the subject indicate that this outcome is a complex, dynamic concept that involves a state of greater vulnerability to adverse health factors, including those that are associated with falls, fractures, disability and health status [1,2,3], and that frailty increases the chances of morbidity and mortality [4].

Limiting or cancelling imminent risk factors and increasing protective factors are potential actions to reduce the frailty condition [5]. Increased age, chronic diseases, low socioeconomic status, inadequate nutrition and low levels of physical activity are risk factors usually associated with frailty [6], and some of these components are used as criteria to determine the frailty syndrome.

Regular engagement in moderate-to-vigorous physical activity has been shown to be a protective behaviour against frailty in the elderly [7,8]. However, studies indicate that exacerbated exposure to sedentary behaviour—activities performed in a sitting/reclining position that do not substantially increase energy expenditure above resting values ≤1.5 METs [9]—are associated with a higher propensity to adverse health factors [10,11,12], even in sufficiently active individuals [13].

Human ageing usually leads to reductions in physical activity level [14,15] and increased time spent in sedentary behaviours [16,17,18]. Prolonged sitting/lying time is a behavioural aspect that may exacerbate or even trigger the frailty syndrome process in older adults [11].

It is worth mentioning that no studies were found in the literature that determined a prerequisite amount of time spent in sedentary behaviour to predict frailty in elderly people. The elucidation of the amount of time spent in sedentary behaviour necessary to discriminate the state of frailty provides support for the creation of more assertive public policies directed toward the promotion of active lifestyles. The objective of this study was to analyse the relationship between time spent in sedentary behaviour and frailty in older adults.

## 2. Materials and Methods

### 2.1. Characterization of the Study and Population

This is a cross-sectional study of the population-based epidemiological survey *entitled* Longitudinal Study of the Elderly Health of Alcobaça, BA (ELSIA Project). According to the latest census, the population of the municipality was 21,319 inhabitants, with 2047 people aged 60 and over, of whom 1024 represented the total number of older adults living in the urban area of the municipality [19]. A survey with individuals registered in the Family Health Strategy (FHS) of the Health System of the Brazilian government was conducted in the municipality of Alcobaça, located in the extreme south of the state of Bahia, north-eastern Brazil. The municipality of Alcobaça has 743 older adults enrolled in the FHS; of these, 54 refused to participate in the survey, 58 were excluded because they did not meet the inclusion criteria and 158 were not located, resulting in a final sample of 473 individuals aged 60 years or older. Individuals were excluded from the study if they had a score of <12 points on the Mini-Mental State Examination [20]; an inability to ambulate, even with the assistance of a cane or walker; severe difficulty in visual and auditory acuity, according to the interviewer’s perception; wheelchair dependence; and severe sequelae of cerebrovascular accident with localized loss of strength.

### 2.2. Data Collection Procedures

Two physical performance tests were used to assess upper limb strength and gait speed. Anthropometric measures (weight, height) were used to calculate body mass index (BMI) in kg/m². Data on sociodemographic aspects, health indicators and behavioural aspects were obtained using a home-based survey and individual interview. The data collection team was composed of previously trained health professionals.

#### 2.2.1. Frailty Syndrome

Frailty was classified according to the five criteria proposed by Fried et al. [2]: (1) Unintentional weight loss; (2) Exhaustion assessed by self-report of fatigue; (3) Muscle weakness; (4) Slowness evaluated by walking speed; (5) Insufficient level of physical activity. Unintentional weight loss was assessed by the following question: “In the past year, did you lose more than 4.5 kg unintentionally (i.e., no diet or exercise)?” If the answer was yes, the participant fulfilled the criterion for frailty in this item by computing one point.

Exhaustion was defined based on two questions from the Geriatric Depression Scale—short form, validated for the Brazilian population: “Did you stop doing many of your activities and interests?” and “Do you feel energetic?” [21]. A positive answer to the first question and/or a negative answer to the second question were considered as signs of exhaustion/fatigue, and a point was computed as a criterion of frailty.

Muscle weakness was assessed by palmar gripping force using a SAEHAN type dynamometer (Saehan Corporation SH5001, Yangdeok-Dong, Masan, South Korea). The test procedure followed the recommendations of the American Society of Hand Therapists (ASHT): participants performed the test while sitting in a chair, with a shoulder adducted, the elbow flexed at 90°, the forearm in a neutral position, and the wrist between 0° and 30° in extension; the movable handle in position II for women and in position III for men, where the test was performed with a verbal stimulus of 6 s. Three measures, in kilograms-force (kgf) were obtained for the dominant hand and the average value of the three measures was determined. The cut-off points, adjusted for sex and BMI, were those proposed by Fried et al. [2].

Slowness was determined by walking speed (WS) during a walk test of 4.57 m. Slowness was adjusted according to sex and height. The cut-off points for frailty were 7 s and 6s for men with height ≤173 cm and men taller than 173 cm, respectively. For women, the cut-off points were also 7 s and 6s but for heights ≤159 cm and >159 cm, respectively.

Physical inactivity was determined using the long version of the International Physical Activity Questionnaire (IPAQ) validated for the Brazilian elderly population [22,23]. The IPAQ asks questions related to moderate to vigorous intensity physical activity performed in a usual week in four domains: work, transportation, domestic activities and leisure activities. Physical inactivity was characterized considering the minimum recommendations for physical activity practice of ≥150 min/week or 75 min of vigorous activity × 2. Thus, <150 min/week was adopted as a cut-off point for the insufficient level of moderate to vigorous intensity physical activity [24].

The scores from the abovementioned tests were summed to generate a total score, which was classified on an ordinal scale from 0 to 5 points. Presence of frailty was defined as a score of ≥3 points [2]. Based on this criterion, 457 individuals were classified as frail and were included in the analysis.

#### 2.2.2. Sedentary Behaviour

Sedentary behaviour was evaluated using time spent sitting during a typical weekday and weekend day (min/day), according to the IPAQ. The following equation was applied to calculate average time spent sitting per day: [(Week × 5) + (weekend × 2)]/7 [25].

#### 2.2.3. Sociodemographic Variables

The sociodemographic variables included in the survey were age range (60–69, 70–79 and 80 years or more); gender (male, female); years of study (>4 years, ≤4 years); and family arrangement (live alone, accompanied).

#### 2.2.4. Health Indicators

The health indicators collected in this study were health perception (positive, negative); functional impairment defined as the presence of at least one limitation for performing basic activities of daily living (BADL) and instrumental activities of daily living (IADL). The BADLs were evaluated by the Katz Index and were reported on six questions related to self-care activities, such as: Bathing, dressing, going to the toilet, lying down and getting out of bed, eating and controlling the physiological elimination functions (urinating and/or defecating) [26]. The IADLs were assessed using the Brazilian version of Lawton’s scale [27], consisting of seven questions related to independence in maintenance activities, such as: Using the telephone, going to distant places using transportation, shopping, preparing their own meal, cleaning and organising the house, taking medicines and handling finances.

### 2.3. Statistical Procedures

Data were doubly entered in the Epidata software, version 3.1b. Statistical analyses were performed in the Statistical Package for Social Sciences version 21.0 (SPSS Inc., Chicago, IL, USA), and MedCalc software version 16.2.1 (MedCalc Software bvba, Ostend, Belgium). Descriptive data were presented as mean and frequency, and the Chi-square test was used for comparing variables according to gender.

Sensitivity and specificity values, as well as the respective cut-off points of time spent in sedentary behaviour for predicting frailty were identified through receiver operating characteristic (ROC) curves. The total area under the ROC curve between sitting time and presence of frailty was identified. The greater the area under the ROC curve, the greater the discriminatory power of sitting time for the presence of fragility. A 95% confidence interval (CI) was used. 

To determine if time spent in sedentary behaviour higher than the cut-point value may be indicative of higher likelihood of frailty, logistic regressions were conducted in two steps: (1) in the first step, crude logistic regression was performed between frailty and sociodemographic information, health indicators and sedentary behaviour; (2) adjusted odds ratios were calculated between frailty and sedentary behaviour cut-points for those sociodemographic and health indicators that were significant (*p* < 0.20) in the crude analysis. A level of significance set at 5% and 95% confidence intervals (95% CI) was calculated in all analyses.

### 2.4. Ethical Procedures

This research followed the ethical principles of the Declaration of Helsinki and Resolution no. 466/12 of the National Health Council. The research protocols were evaluated and approved by the Research Ethics Committee of the Federal University of Triangulo Mineiro (Ruling No. 966/2015).

## 3. Results

The study population consisted of 285 women (62.4%) and 172 men (37.6%). Ages ranged from 60 to 97 years, with a mean age of 70.25 years (SD = 8.25). The prevalence of frailty was 22.1%(*n* = 101). In relation to the five components of frailty analysed, 21.0% (*n* = 96) presented unintentional weight loss, 31.5% (*n* = 144) exhaustion, 49.7% (*n* = 227) muscle weakness, 8.5% (*n* = 39) slowness, 46.2% (*n* = 211) insufficient level of physical activity. The median value (25th, 75th percentile) for sedentary behaviour was 418.57 (322.50, 528.21) min/day.

Other characteristics of the population, according to presence of frailty are shown in Table 1. The frequency of frailty was significantly different between age groups, schooling and, BADL and IADL.

Table 2 shows the areas under the ROC curves with their respective confidence intervals, sensitivity and specificity for time spent in sedentary behaviour as a predictor of frailty. ROC curves were constructed for males and females. The largest area was observed for males (0.74).

**Figure 1:**
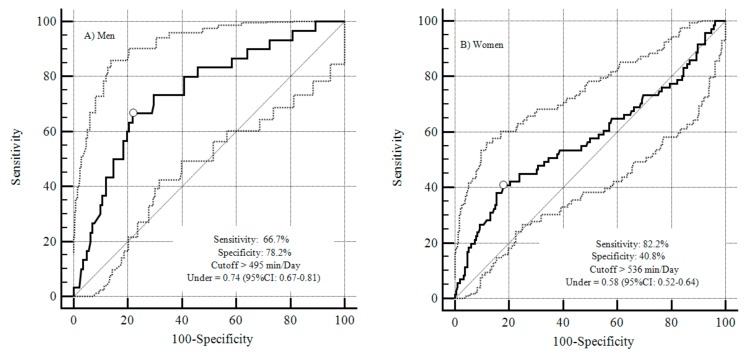
Mean daily time exposed to sedentary behaviour as a discriminant criterion for frailty in the elderly. Solid line: Area under the curve limit; Dotted line: Confidence interval of the area under the curve. (**A**) Men; (**B**) Women.

The cut-off points of the mean daily time spent in sedentary behaviour as a discriminant for frailty can be visualized in Figure 1. For males, the cut-off point for sedentary behaviour was >495 min/day and for women >536 min/day.

Table 3 shows the crude and adjusted associations between frailty and sociodemographic information, health indicators and sedentary behaviour. Those individuals with time spent in sedentary behaviour greater than the values defined in the ROC curves were more likely to present frailty (OR 3.01; 95% CI: 1.78–5.10) than those who were sitting less. This estimate was adjusted for gender, age, years of schooling, perception of health, BADL and IADL.

## 4. Discussion

The present study aimed to analyse the time spent in sedentary behaviour as a discriminant criterion for frailty in older adults. Frailty represents a complex syndrome characterized as an important construct for the diagnosis of the health of older adults. The diagnosis of frailty allows practitioners to identify the decline in physical function of older adults and, consequently, to identify adverse health factors. In addition, the evaluation of sedentary behaviour provides complementary information on the health of older adults, regarding healthy ageing and adverse health factors [17,28]. Sedentary behaviour is an imminent risk factor that has gained attention in the literature. It is a consensus that this behavioural aspect is associated with deleterious health effects [29,30], including risk for all-cause mortality [4,31] and frailty [11].

Approximately one-fifth of the participants from this study were frail. The prevalence of frailty in older adults living at poverty and social vulnerability in Brazil is 27.3%, with an additional pre-frailty prevalence of 60.6% [32]. The high occurrence of frailty in Brazilian older adults highlights the importance of greater assistance in health care, as well as its socioeconomic costs [33].

In the present study, the relationship between sedentary behaviour and frailty in both sexes was observed. These results corroborate the findings of recent investigations [34,35,36]. This fact confirms sedentary behaviour as a behavioural aspect that may determine the state of frailty and it is consequently an adverse health behaviour in the elderly [28]. Increased time spent in the seated position results in the increased exposure of the body to inflammatory and disabling conditions, which in turn raises the probability of frailty in older individuals [37].

In this study, the cut-off points of sedentary behaviour for predicting frailty were >495 min/day (men) and >536 min/day (women). The areas under the curves for sedentary behaviour indicate a larger area for men compared to women. On the other hand, women may need more exposure time to sedentary behaviours in order to experience significantly greater chances of frailty [35]. It is important to highlight that the latter result was found in a hospital setting; thus, reverse causality cannot not be ruled out. 

The cultural aspects that involve differences in activities normally performed by men and women in Brazil and the ageing process itself may explain the results found in this study. In general, when men retire, they tend to spend more time in a sitting/reclining position and less time in physical labour activities. In certain cases, they may experience greater functional impairment and reliance on their spouse [18,38]. On the other hand, although older women also tend to spend more time in a sitting/reclining position after retiring, such a factor is offset by their daily routine, which includes domestic tasks that substantially contribute to their physical activity level [16,17,39]. Despite this fact, the results of this study indicated that women had a significantly higher odds for frailty compared to men (OR: 1.91 (95% CI: 1.10–3.31)).

The association between sedentary behaviour and frailty is still not well explored. The scarcity of recommendations for cut-off points for sedentary behaviour, as well as methodological differences, make it difficult to compare the results of this study with those of other studies. It is understood that the longer the exposure time to sedentary behaviour, the greater the vulnerability to frailty [11] and adverse health factors [28].

The harm of sedentary behaviour to health and longevity obtains consensus in the literature [37]. A balance between active and sedentary behaviours is necessary. The cut-off points set forth in this study may serve to fill a gap in the literature regarding the time exposed to sedentary behaviour that can discriminate frailty in older adults of both sexes. The cut-off points for sedentary behaviour described in this study represent only reference estimates for use in the screening process in population interventions aiming at preserving functionality in older adults. Prospective studies in different regions are needed to identify sedentary behaviour exposure time that can discriminate in affecting frailty levels in other cultural contexts.

This study provides a simple method for early identification of older adults that may be at an increased likelihood of becoming frail. Our results may be used in the clinical setting to screen those individuals that may need special attention for avoiding frailty. Future studies should examine the validity of the cut-points in different samples and also seek to understand if the association of sedentary behaviour and frailty is a causal one.

One of the limitations of the study include the cross-sectional design that prevents assessment of the cause and effect relationship between the variables; however, cross-sectional studies do allow for making inferences concerning the observed results. Additionally, the use of a self-report questionnaire may have underestimated or overestimated some estimates, especially regarding education and the motivational aspects of participants. However, the study assessors underwent training prior to data collection to minimize motivational interference and to standardize the instructions during the interview. Among the strengths of the study were the relatively large sample and the controlling of several confounding factors, which allowed for better identification of the associations of sedentary behaviour with presence of frailty.

## 5. Conclusions

The results of this study indicate that the average daily time exposed to sedentary behaviour may predict the presence of frailty in older adults. Regarding the amount of time needed to discriminate the presence of frailty, the results from this study indicated that >495 min/day and >536 min/day of sedentary behaviour predicted frailty in older men and women, respectively, in this study.

## Figures and Tables

**Table 1:** Participants characteristics according to frailty classification.

Variable	Non-Frail% (*n*)	Frail% (*n*)	χ^2^ (*p*)
**Age group (years)**			
60–69	62.6 (*n* = 223)	30.7 (*n* = 31)	* 47.87
70–79	28.1 (*n* = 100)	34.7 (*n* = 35)	(<0.001)
≥80	9.3 (*n* = 33)	34.7 (*n* = 35)	
**Sex**			
Male	39.9 (*n* = 142)	29.7 (*n* = 30)	* 3.47
Female	60.1 (*n* = 214)	70.3 (*n* = 71)	(0.062)
**Years of schooling**			
>4 years	35.5 (*n* = 126)	19.0 (*n* = 19)	* 9.77
≤4 years	64.5 (*n* = 229)	81.0 (*n* = 81)	(0.002)
**Home arrangement**			
Lives alone	16 (*n* = 57)	16.8 (*n* = 17)	*0.039
Accompanied	84 (*n* = 299)	83.2 (*n* = 84)	(0.843)
**Perception of health**			
Positive	38.8 (*n* = 138)	29.7 (*n* = 30)	* 2.77
Negative	61.2 (*n* = 218)	70.3 (*n* = 71)	(0.096)
**BADL**			
Independent	82.6 (*n* = 294)	64.4 (*n* = 65)	* 15.51
Dependent	17.4 (*n* = 62)	35.6 (*n* = 36)	(<0.001)
**IADL**			
Independent	34.3 (*n* = 122)	11.9 (*n* = 12)	* 19.03
Dependent	65.7 (*n* = 234)	88.1 (*n* = 89)	(<0.001)

* Pearson’s chi-square; BADL = Basic activities of daily living; IADL = Instrumental activities of daily living.

**Table 2:** Areas under the ROC curve, confidence interval (95% CI) and the respective values of sensitivity and specificity of the time exposed to sedentary behaviour as a predictor of frailty in older adults.

	Area under the ROC Curve	95% CI	Sensitivity	Specificity
Men	0.74	0.67–0.81	66.7	78.2
Women	0.58	0.52–0.64	82.2	40.8

**Table 3:** Association of sociodemographic variables, health indicators and sedentary behaviour with frailty.

Variable	Frailty			
	CrudeAnalysis		Adjusted Analysis	
	OR (95% CI)	*p*-Value	OR (95%CI)	*p*-Value
**Gender**		0.063		0.021
Male	1		1	
Female	1.57 (0.97–2.53)		1.91 (1.10–3.31)	
**Age group (years)**		<0.001		<0.001
60–69	1		1	
70–79	2.52 (1.47–4.31)		2.17 (1.22–3.84)	
≥80	7.63 (4.16–13.99)		5.57 (2.87–10.80)	
**Years of schooling**		0.002		0.120
>4 years	1		1	
≤4 years	2.35 (1.36–4.04)		1.64 (0.88–3.07)	
**Home arrangement**		0.843		-
Lives alone	1		-	
Accompanied	0.94 (0.52–1.70)		-	
**Perception of health**		0.097		0.920
Positive	1		1	
Negative	1.50 (0.93–2.41)		1.03 (0.60–1.77)	
**BADL**		<0.001		0.045
Independent	1		1	
Dependent	2.62 (1.61–4.29)		1.78 (1.01–3.13)	
**IADL**		<0.001		0.169
Independent	1		1	
Dependent	3.87 (2.04–7.34)		1.67 (0.80–3.48)	
**Sedentary Behaviour (min/Day)**		<0.001		<0.001
≤495 for males and ≤536 for female	1		1	
>495 for males and >536 for female	3.85 (2.41–6.16)		3.01 (1.78–5.10)	

BADL = Basic activities of daily living; IADL = Instrumental activities of daily living.
